# Spermine Attenuates the Action of the DNA Intercalator, Actinomycin D, on DNA Binding and the Inhibition of Transcription and DNA Replication

**DOI:** 10.1371/journal.pone.0047101

**Published:** 2012-11-08

**Authors:** Sheng-Yu Wang, Yueh-Luen Lee, Yi-Hua Lai, Jeremy J. W. Chen, Wen-Lin Wu, Jeu-Ming P. Yuann, Wang-Lin Su, Show-Mei Chuang, Ming-Hon Hou

**Affiliations:** 1 Department of Life Science, National Chung Hsing University, Taichung, Taiwan; 2 Institute of Genomics and Bioinformatics, National Chung Hsing University, Taichung, Taiwan; 3 National Institute of Cancer Research, National Health Research Institutes, Miaoli, Taiwan; 4 Institute of Biomedical Sciences, National Chung Hsing University, Taichung, Taiwan; 5 Department of Biotechnology, Ming Chuan University, Taoyuan County, Taiwan; University of Helsinki, Finland

## Abstract

The anticancer activity of DNA intercalators is related to their ability to intercalate into the DNA duplex with high affinity, thereby interfering with DNA replication and transcription. Polyamines (spermine in particular) are almost exclusively bound to nucleic acids and are involved in many cellular processes that require nucleic acids. Until now, the effects of polyamines on DNA intercalator activities have remained unclear because intercalation is the most important mechanism employed by DNA-binding drugs. Herein, using actinomycin D (ACTD) as a model, we have attempted to elucidate the effects of spermine on the action of ACTD, including its DNA-binding ability, RNA and DNA polymerase interference, and its role in the transcription and replication inhibition of ACTD within cells. We found that spermine interfered with the binding and stabilization of ACTD to DNA. The presence of increasing concentrations of spermine enhanced the transcriptional and replication activities of RNA and DNA polymerases, respectively, *in vitro* treated with ActD. Moreover, a decrease in intracellular polyamine concentrations stimulated by methylglyoxal-bis(guanylhydrazone) (MGBG) enhanced the ACTD-induced inhibition of c-myc transcription and DNA replication in several cancer cell lines. The results indicated that spermine attenuates ACTD binding to DNA and its inhibition of transcription and DNA replication both *in vitro* and within cells. Finally, a synergistic antiproliferative effect of MGBG and ACTD was observed in a cell viability assay. Our findings will be of significant relevance to future developments in combination with cancer therapy by enhancing the anticancer activity of DNA interactors through polyamine depletion.

## Introduction

The binding of many important anticancer drugs or antibiotics to DNA plays an important role in their chemotherapeutic functions [1]. These drugs are thought to exert their primary clinical effects via interference with DNA function by blocking DNA replication and gene transcription [2]. Significant insights into DNA conformation and drug-DNA interactions for the design of future useful drugs were provided by studies of the three-dimensional structures of several DNA-antitumor drug complexes [3]–[6]. Two classes of noncovalent DNA binding drugs, intercalators and groove binders, have been identified. Intercalators, such as actinomycin D (ACTD), bind to DNA by inserting a planar aromatic chromophore between adjacent DNA base pairs [7], [8]. The biological activity of ACTD is related to its ability to bind to the DNA duplex with high affinity, thereby interfering with replication and transcription [9], [10].

Polyamines, such as spermine, spermidine, and putrescine, were demonstrated to be involved in cell growth and differentiation [11], [12]. The levels of polyamines in cells, especially in the nucleus, are detected in the millimolar (mM) range [11]. Polyamine metabolism is frequently dysregulated in cancer cells and is associated with higher polyamine concentrations than those observed in normal cells [13]. The inhibition of polyamine biosynthesis by polyamine inhibitors is a potential strategy for cancer chemotherapy [14]. Polyamines carry multiple positive charges (*e.g.*, four in spermine) at physiological pH values because of the protonation of the amine groups. Polyamines, particularly spermine, are almost exclusively bound to nucleic acids via nonspecific electrostatic interactions and hydrogen bonds [15], [16]. Previous studies have shown that the spermine binding sites in DNA are located within both the major and minor grooves [17]–[19]. The binding of spermine to DNA eliminates the difference in stability among various DNA structures, which then allows them to be inter-converted with minimal energy barriers [20]. Moreover, spermine has been shown to be involved in many cellular processes that require nucleic acids; for example, it regulates transcription by affecting the binding ability of RNA-binding proteins [16], [21], and it interacts with DNA to promote DNA condensation, which protects the bound DNA against denaturation and DNA damaging agents, such as ionizing radiation and reactive oxygen species (ROS) [22]–[28]. Kumar et al. have found that polyamines modulate the conformation of G-quadruplex in the promoter region and further affect the downstream gene expression [29]. We previously discussed the effects of polyamines on the DNA-binding properties of the metal derivative complex of aureolic family drugs [30]. Nevertheless, the effects of polyamines on the action of DNA intercalators remain unclear because intercalation is the most important binding mode for DNA-binding drugs [7], [8].

In this study, using ACTD as a model, we conducted a systematic study to determine the effects of spermine on the DNA-acting properties of ACTD, including its DNA-binding ability and the inhibition of DNA replication and transcription both *in vitro* and within cells. We observed that the action of ACTD on DNA *in vitro* is attenuated by spermine. Decreasing intracellular polyamine levels enhanced the inhibition of ACTD on c-myc transcription, DNA replication, and cell viability in several cancer cell lines. This work provides insight into the role of polyamine-DNA interaction in affecting the anticancer properties of a DNA intercalator, suggesting that the combination of DNA intercalators and polyamine inhibitors might be an effective anticancer strategy.

## Materials and Methods

ACTD, methylglyoxal-bis(guanylhydrazone) (MGBG), and spermine were purchased from Sigma Chemical Co. (St. Louis, MO). Absorbance measurements were conducted using a quartz cuvette and a Hitachi U-2000 spectrophotometer. The concentration of ACTD was estimated using an extinction coefficient of 35,280 M^−1^cm^−1^ at 224 nm [31]. The concentrations of oligonucleotides were determined according to Beer's law (A = ε·b·c, A: optical density at 260 nm; ε: extinction coefficient; b: cell path length, 1 cm; c: DNA concentration in M). Synthetic DNA oligonucleotides were purified by gel electrophoresis. Oligomer extinction coefficients were calculated according to tabulated values of monomer and dimer extinction coefficients, with reasonable assumptions [32].

### Circular dichroism (CD) experiments

CD spectra were collected between 520 and 200 nm at 1-nm intervals using a JASCO-815 spectropolarimeter. Temperature was controlled by a circulating water bath. All spectra were calculated as the average of three runs. The methods used for the CD spectral analyses have been described previously [33]. The molar ellipticity [*θ*] was calculated from the equation [*θ*] = *θ*/*Cl*, where *θ* is the relative intensity, *C* is the molar concentration of oligonucleotides, and *l* is the path length of the cell in centimeters.

### Thermodynamic parameters measurements

The UV absorbance vs. temperature profiles were measured using a JASCO V560 UV/VIS spectrophotometer to monitor the sample absorption (OD) at 260 nm. The sample cell was equipped with a Peltier-type cell holder (EHC-441), and the temperature was regulated by a programmer (JASCO TPU-436). The concentration of duplex DNA in each sample was 4 µM in a 20 mM sodium cacodylate buffered solution at pH 7.3. The experiments were performed by increasing the temperature from 5 to 95°C at a rate of 0.5°C/min, and the temperature was recorded every 30 sec. The data sets from each melting curve were normalized to minimize the variations in each experiment because *T*_m_ is independent of DNA concentration. To obtain van't Hoff transition enthalpies, the UV melting curves were evaluated and the experimental absorbance vs. temperature curve was converted into a melted fractions vs. temperature curve. The melted fractions in the single strands (*f*) vs. temperature (*T*) plots were calculated by fitting the melting profile to a two-state transition model [34]. The *T*_m_ were evaluated directly from the temperature at *f* = 0.5. The thermodynamic parameters of DNA with and without ACTD in the presence of spermine at various concentrations were estimated using the melting profiles according to a previously described method [35], [36].

### Surface plasmon resonance (SPR) binding analysis

The affinity between the drug and DNA duplexes was measured in a BIAcore 3000A SPR instrument (Pharmacia, Uppsala, Sweden) with a SensorChip SA5 (Pharmacia) by monitoring the refractive index change of the sensor chip surface. These changes are generally assumed to be proportional to the mass of the molecules bound to the chip and are recorded in resonance units (RU). The 5′-biotin-labeled hairpin DNA duplexes, 5′-AAAGCTTTTGTAAAGCTTT-3′ used in the SPR experiments were purified by PAGE. To control the amount of DNA that bound to the streptavidin SA chip surface, the biotinylated oligomer was manually immobilized on the chip surface. ACTD was prepared in a solution of 20 mM Tris–HCl (pH 7.3), 50 mM NaCl, and various concentrations of polyamines. Different concentrations of the drug were passed over the chip surface for 180 sec at a flow rate of 70 µl min^−1^ to reach equilibrium, and one of the flow cells was kept blank as a control. The blank buffer solution was then passed over the chip to initiate the dissociation reaction, and this flow was continued for an additional 300 sec to complete the reaction. The surface was then recovered by washing with 10 µl of 10 mM HCl. Analyses of the SPR-binding constants have been described previously [20]. Sensorgrams for the interactions between the hairpin DNA duplexes and drugs were analyzed using BIA evaluation software, version 3. The fit was accepted when the chi-square values were less than 3.

### RNA polymerase assays

To analyze *in vitro* transcription, a T7 RNA polymerase assay (Promega) was used. The primer-template complex included linearized DNA template (pTRI-β-actin-mouse,) and T7 primer. The primer-template complex with or without ACTD was heated at 95°C for 5 min and equilibrated at 4°C for 10 min in reaction buffer (40 mM Tris, pH 7.8, 20 mM NaCl, 2 mM spermidine, and 0.1% of β-ME). The enzyme plus NTP reaction solution was micropipetted into the DNA-ACTD complex solution in the presence of spermine at various concentrations and incubated at 37°C for 60 min and digested with 1 µl of TURBO DNase to remove DNA at 37°C for 15 min. The final enzyme and NTP concentrations were 2 U and 1 mM, respectively. An aliquot (10 µl) of the reaction buffer was removed and quenched by adding loading buffer (95% formamide, 0.025% xylene cyanol, 0.025% bromophenol blue, 18 mM EDTA, 0.025% SDS) and heating at 80°C for 5 min. The reaction products were examined by native gel electrophoresis at 50 V in 10% polyacrylamide (10 cm×10.5 cm×0.75 mm) in TBE buffer. The gels were stained with SYBR Green, and the bands were detected in the gel using Imagemaster VDS (Pharmacia).

### DNA polymerase assays

*E. coli* DNA polymerase I was used for the DNA polymerase assay (Promega). For the polymerization reactions, the DNA template (5′-TGTTGCATAGCAGTCACAGGTCAGGCTAGCTGGGCAAGAACTGGCA-3′) and primer (5′-TGCCAGTTCTTGCCCAGCTAG-3′) from the cdc7 gene were used. Primer-template duplexes with or without ACTD were heated at 95°C for 5 min and equilibrated at 4°C for 10 min in reaction buffer (10 mM Tris-HCl, pH 7.5, 7 mM MgCl_2_, and 0.1 mM DTT). The enzyme plus dNTP reaction solution was micropipetted into the DNA-ACTD complex solution in the presence of spermine at various concentrations and incubated at 37°C for 10 min. The final enzyme activity and dNTP concentrations were 0.5 U and 2 mM, respectively. An aliquot (10 µl) of the reaction buffer was removed and quenched by adding loading buffer (50% glycerol, 0.25% bromophenol blue, and 0.25% xylene cyanol) and heating at 80°C for 10 min. The reaction products were examined by denaturing gel electrophoresis at 100 V in 10% polyacrylamide (10 cm×10.5 cm×0.75 mm) in TBE buffer, with 12 M formamide as a denaturant. The gels were stained with SYBR Green, and the bands were detected in the gel using Imagemaster VDS (Pharmacia).

### Intracellular polyamine concentration determination

The methods used for the polyamine content analysis have been described previously [30]. Intracellular polyamines were extracted with 3% perchloric acid (v/v). The supernatant was subjected to dansylation with dansyl chloride in acetone. The dansyl amides were extracted with toluene and separated by thin layer chromatography (TLC) using ethyl acetate/cyclohexane (2∶3, v/v) as the mobile phase. The intensity was quantified by a spectrophotometer with excitation and emission at 365 and 505 nm, respectively.

### Reverse transcription and real-time PCR

The human cervical carcinoma (HeLa) cell was grown in DMEM medium containing 10% fetal bovine serum, 1% L-glutamine, and 1% penicillin/streptomycin and maintained at 37°C in a humidified atmosphere containing 5% CO_2_ and 95% air. The human bronchial epithelial cell (BEAS-2B), the human lung adenocarcinoma cell (A549) and the human breast adenocarcinoma cell (MCF7) were cultured in RPMI medium with 10% foetal bovine serum containing 1% penicillin/streptomycin at 37°C in a humidified atmosphere of 5% CO_2_. The cells were seeded on 6-well culture plates at a density of 1×10^6^ cells. The cells were then incubated in the presence or absence of 2 µM MGBG for 24 h. Thereafter, the MGBM-containing solution was washed out and replaced with a solution containing the specified concentration of ACTD for another 6 h. Total RNA was isolated from cells using an Illustra RNA extraction kit. The concentration of total RNA was determined by the absorbance at 260 nm. To prepare a cDNA pool from each RNA sample, 5 µg of total RNA was reverse transcribed using MMLV reverse transcriptase (Promega), and the resulting samples were diluted 40 times with DNase-free water. Each cDNA pool was stored at −20°C until real-time PCR analysis. The real-time PCR reactions were performed on a Roche LightCycler Instrument 1.5 using the LightCycler® FastStart DNA Master^PLUS^ SYBR Green I kit (Roche Cat. 03 515 885 001, Castle Hill, Australia). Briefly, each 10 µl reaction contained 2 µl Master Mix, 2 µl each of 0.75 µM actin forward primer and 0.5 µM c-myc reverse primer, and 6 µl cDNA sample. The primer sequences for actin and c-myc were described previously [37]. Each sample was run in triplicate. The RT-PCR program used was 95°C for 10 min, followed by 40 cycles of 95°C for 10 sec, 60°C for 15 sec, and 72°C for 10 sec. At the end of the program, a melting curve analysis was performed. At the end of each RT-PCR run, the data were automatically analyzed by the system, and an amplification plot was generated for each cDNA sample. From each of these plots, the LightCycler3 Data analysis software automatically calculated the CP value (crossing point, or the turning point corresponding to the first maximum of the second derivative curve), which represents the beginning of exponential amplification. The fold expression or repression of the target gene relative to the internal control gene (actin) in each sample was then calculated.

### BrdU incorporation assay

HeLa cells were seeded into 96-well culture plates at a density of 1×10^4^ cells. The cells were incubated in the presence or absence of 2 µM MGBG for 24 h. Thereafter, the MGBM-containing solution was washed out and replaced with a solution containing the indicated concentration of ACTD for another 24 h. DNA replication was assessed by examining 5-bromo-2′-deoxyuridine (BrdU) incorporation into cellular DNA during cell proliferation using an anti-BrdU antibody in cultured cells. BrdU labeling was performed using the BrdU incorporation ELISA kit from Roche Applied Sciences according to the manufacturer's instructions.

### Cell viability assay

Cells were seeded into 96-well culture plates at a density of 1×10^4^ cells. Cell viability was evaluated by the MTT (dimethylthiazol diphenyltetrazolium bromide: thiazolyl blue) assay. The cells were incubated in the presence or absence of 2 µM MGBG for 24 h. Thereafter, the MGBM-containing solution was washed out and replaced with a solution containing the indicated concentration of ACTD for another 24 h. Subsequently, the MTT solution (500 µg/ml medium, final concentration) was added to each well, and the cells were then incubated at 37°C for 2 h. The solution was removed, and isopropanol was added to solubilize the stain. The results were evaluated using a SpectraMax M2 plate reader (Molecular Devices, Sunnyvale, CA), which yielded the absorbance intensity as a function of the number of viable cells.

### Statistical analysis

The cell assay results were expressed as the standard error of the mean (S.E.M.), S_X_ = S/n^1/2^, where S is the standard deviation and n is the number of experiments. The mean values were compared using two-way analysis of variance (ANOVA) (SigmaStat v.2, SPSS Inc., Chicago, IL). The level set for statistical significance was *p*<0.05.

## Results

### The effects of spermine on the DNA-binding affinity of actinomycin D bound to hairpin DNA duplexes

**Figure 1:**
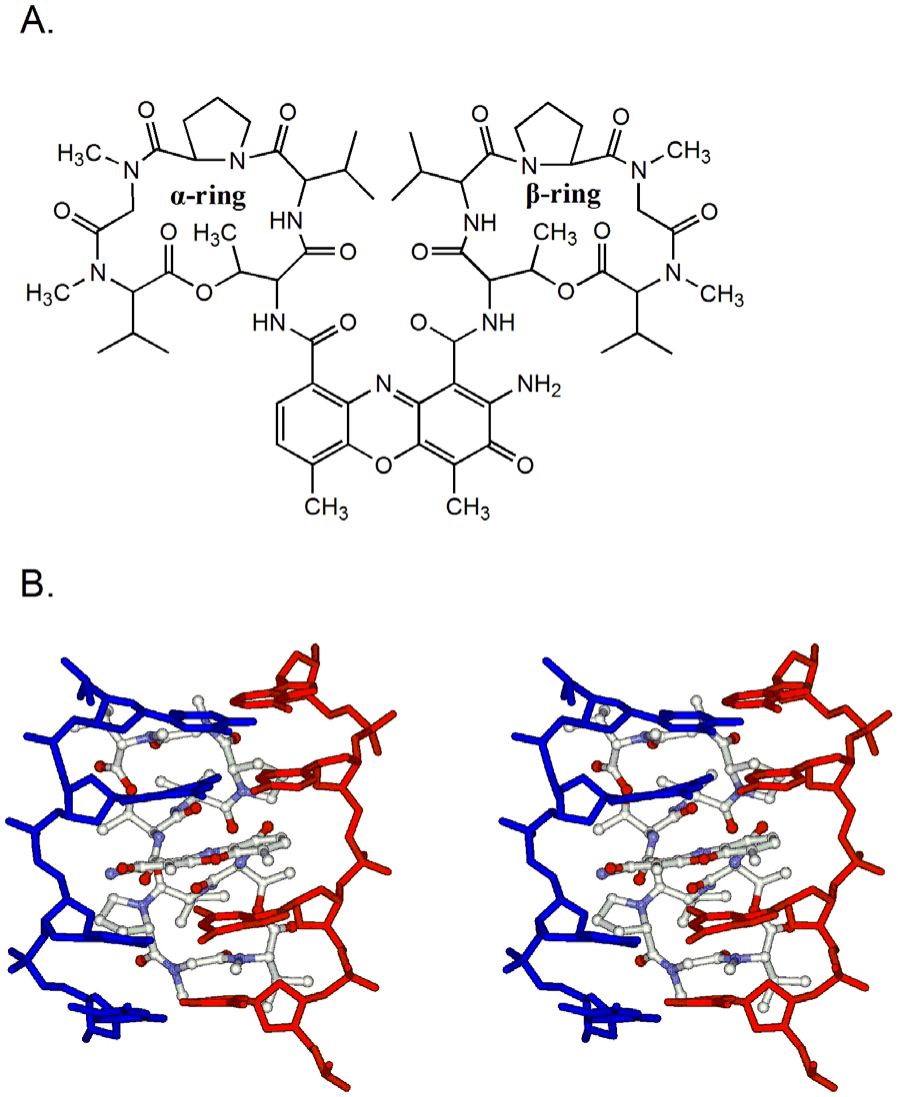
The binding of actinomycin D to DNA. (**A**) The chemical structure of actinomycin D (ACTD). (**B**) Magnified side stereoview of the ACTD-TGCA sequence complex interface (ACTD as a ball-and-stick representation and DNA as a skeletal representation) (PDB:1MNV). The phenoxazone ring is intercalated individually into the GC step.

**Figure 2:**
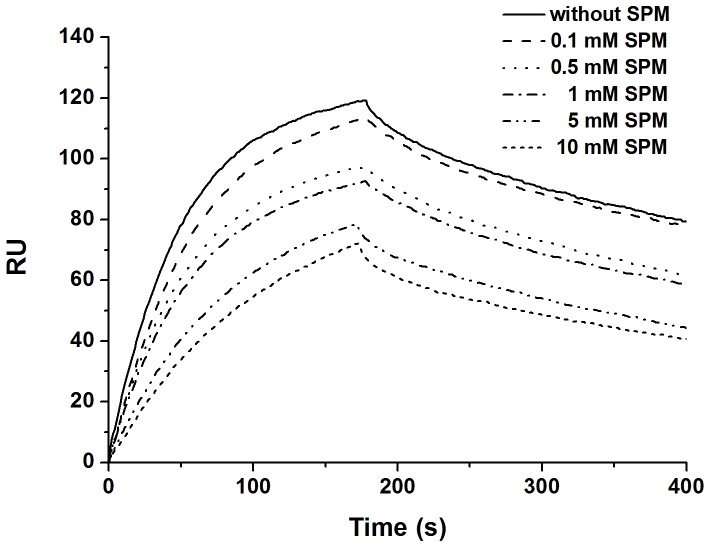
The effects of spermine on the DNA-binding affinity of actinomycin D. Sensorgrams of the interaction between an immobilized hairpin duplex and the target ACTD (6 µM) in the presence of various concentrations of spermine (SPM).

ACTD, a typical DNA intercalator, is a potent anticancer drug (Figure 1A). It binds to DNA by intercalating its phenoxazone ring into GpC sequences in the minor groove of DNA with its two cyclic pentapeptides (Figure 1B). The GpC sequence specificity of ACTD is due to the strong hydrogen bonds between the NH/C = O groups of threonines in ACTD with the corresponding N3/N2 sites of the adjacent guanine bases in the GpC step (Figure 1B) [38], [39]. To characterize the effects of polyamines on the binding affinity of ACTD to DNA, ACTD was allowed to interact with biotin-labeled hairpin DNA duplexes in the presence of spermine at various concentrations, and the maximum binding capacity (R_max_) (in RU) was measured by SPR. The biotin-labeled hairpin DNA duplexes provided one ACTD DNA-binding site (AAAGCTTT) with the trinucleotide 5′-TGT-3′ as the loop region. The self-complementary octamer ds(AAAGCTTT) contains a central GpC site, and thus an ACTD molecule is expected to be intercalated at that location. In Figure 2, the association between the hairpin DNA duplexes and ACTD is shown by an increase in the RU values, whereas the dissociation of these two species is shown by a decrease in the same trace. According to the SPR sensorgram, the interactions between the hairpin DNA duplexes and ACTD exhibited the highest maximum capacity (∼120 RU) in the absence of polyamines. Low concentrations of polyamines (0.1 mM) caused minor effects on the DNA-binding affinity of ACTD. The affinity changed by only 8 RU in the presence of 0.1 mM spermine. However, increasing the concentration of spermine to 10 mM significantly decreased the binding capacity of ACTD and DNA, to ∼68 RU.

Kinetic experiments were performed by measuring the parameters of the binding between ACTD and its target DNA duplex with or without polyamines. The kinetic constants of association (*k*_a_ in M^−1^s^−1^) and dissociation (*k*_d_ in s^−1^) for ACTD binding to hairpin DNA duplexes were measured based on the calculations from the association and dissociation phases of the SPR traces, respectively (Table 1). The *k*_a_ value was essentially the same (∼3.19×10^3^ M^−1^s^−1^) at low spermine concentrations of 0.1 and 0.5 mM. Increasing the concentration of spermine to 10 mM decreased the *k*_a_ value of ACTD and DNA. In the presence of 10 mM spermine, the interactions between ACTD and DNA showed a *k*_a_ value of only 1.38×10^3^ M^−1^s^−1^. The dissociation rate constants (*k*_d_) of the buffer alone and 0.1 mM spermine were essentially identical (Table 1). However, the *k*_d_ values for ACTD and DNA increased as the concentration of spermine increased. In the presence of 5 and 10 mM spermine, the interactions between ACTD and DNA reached the highest *k*_d_ value to be, 2.12×10^−3^ and 2.11×10^−3^ M^−1^s^−1^, respectively. These results suggest that spermine affected the binding of ACTD to hairpin DNA duplexes during both the association and dissociation phases. The association constants (*K*_a_) were calculated as *k*_a_/*k*_d_ (in M^−1^). As expected, low concentrations of spermine produced higher *K*_a_ values for the interaction between ACTD and DNA; the *K*_a_ values were approximately three- and four-fold greater than those in the presence of 5 and 10 mM spermine, respectively, showing that high concentrations of spermine significantly reduce the binding affinity of ACTD to hairpin DNA duplexes (Table 1).

**Table 1:** Numerical values of the SPR-derived association rate constants, dissociation rate constants, and association equilibrium constants (*k*_a_, *k*_d_, and *K*_a_) for immobilized hairpin DNA upon binding to ACTD in the presence of various concentrations of spermine.

	*k*_a_ (M^−1^s^−1^)×10^3^	*k*_d_ (s^−1^)×10^−3^	a *K*_a_ (M^−1^)×10^6^
Buffer alone	3.54±0.21	1.43±0.05	2.48±0.17
0.1 mM spermine	3.19±0.15	1.38±0.08	2.31±0.17
0.5 mM spermine	3.15±0.11	1.78±0.10	1.77±0.12
1 mM spermine	2.97±0.08	1.89±0.07	1.57±0.07
5 mM spermine	1.81±0.10	2.12±0.08	0.85±0.06
10 mM spermine	1.38±0.09	2.11±0.06	0.65±0.05

a *Ka* values were obtained by dividing *k_a_* by *k_d_*.

### Effects of spermine on the stabilization of actinomycin D on DNA duplexes

**Figure 3:**
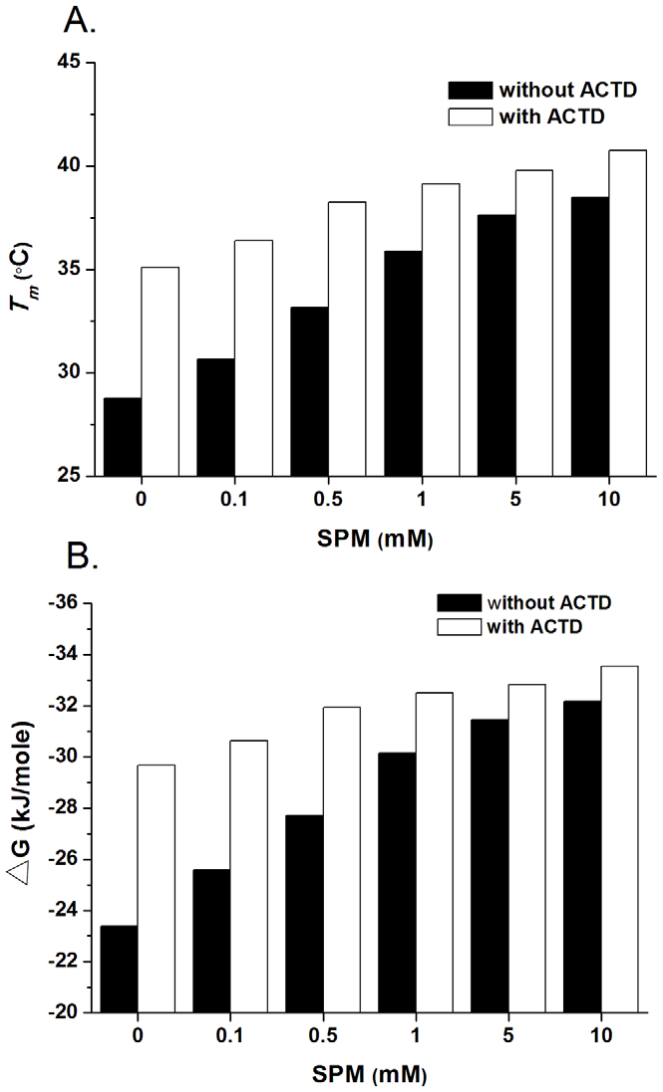
Effects of spermine on the stabilization of actinomycin D on DNA duplexes. (**A**) Melting temperatures (*T_m_* in °C) of DNA in the presence of spermine (SPM) at various concentrations without (filled column) or with (open column) ACTD. The DNA sequence was d(TTTGCAAA). (**B**) Gibb's free energy change, ΔG, for the formation of DNA duplexes in the presence of spermine (SPM) at various concentrations with (open column) or without (filled column) ACTD. Each value was averaged from three separate experimental sets.

To determine the stabilizing effects of ACTD on the formation thermodynamics of DNA duplexes in the presence of various concentrations of spermine, the melting curves of DNA duplexes in the presence of increasing spermine with and without ACTD binding were determined by recording their A_260_ at different temperatures (Figure S1). The *T*_m_ value of the duplex increased by 6.3°C upon the addition of ACTD (Figure 3A), suggesting that intercalation has an important effect on the stability of DNA duplexes. However, the difference is reduced to 5.3°C in the presence of 0.5 mM or 1 mM spermine, and drops sharply upon the addition of 5 or 10 mM spermine. In addition, thermodynamic parameters, such as the Gibb's free energy change (Δ*G*), enthalpy change (Δ*H*), and entropy change (Δ*S*), multiplied by absolute temperature (*T*Δ*S*), were derived from the thermal denaturation of the DNA duplex; the calculated values are listed in Table 2 and 3. The formation of the DNA duplex increased the entropy (Δ*S*) at 298 K with ACTD (Table 3). In addition, the Δ*H* values imply that the formation of the DNA duplex is a less exothermic process in the presence of ACTD. The extent of DNA duplex formation with ACTD is shown using Δ*G* (Figure 3B). The Δ*G* values for the formation of the DNA duplex examined here are negative, showing that their formation is an exergonic process. The Δ*G* for the DNA duplex increased by 6.30 kJ/mol upon the addition of ACTD (Figure 3B). The presence of spermine reduced the difference in Δ*G* observed upon the addition of ACTD; this difference drops sharply, to ∼1.30 kJ/mol, in the presence of 5 or 10 mM spermine. Therefore, in terms of the melting of DNA duplexes, spermine decreased the stabilizing effect of ACTD on the DNA duplex.

**Table 2:** The thermodynamic measurements Δ*G*, Δ*H*, and *T*Δ*S* (in kJ/mol) derived from thermal denaturation for the formation of duplex DNA in the presence of spermine at various concentrations.

	Δ*H*	*T*Δ*S*	Δ*G*
Buffer alone	*−278.3	−254.9	−23.4
0.1 mM spermine	−260.1	−234.2	−25.9
0.5 mM spermine	−264.2	−236.5	−27.7
1 mM spermine	−245.5	−215.3	−30.1
5 mM spermine	−242.3	−210.8	−31.5
10 mM spermine	−240.5	−208.3	−32.2

* each value in this table represents the average of three separate experimental sets.

**Table 3:** The thermodynamic measurements Δ*G*, Δ*H*, and *T*Δ*S* (in kJ/mol) derived from thermal denaturation for the formation of duplex DNA with ACTD binding in the presence of spermine at various concentrations.

	Δ*H*	*T*Δ*S*	Δ*G*
ACTD	−222.7	−193.0	−29.7
0.1 mM spermine	−208.2	−177.5	−30.6
0.5 mM spermine	−220.0	−188.1	−31.9
1 mM spermine	−217.1	−184.6	−32.5
5 mM spermine	−215.5	−182.7	−32.8
10 mM spermine	−213.4	−179.9	−33.6

each value in this table represents the average of three separate experimental sets.

To monitor the effects of spermine on the structure of the DNA duplex during ACTD binding, the interaction between DNA and ACTD was examined with increasing concentrations (0∼10 mM) of spermine by CD spectroscopy (Figure S2). The CD spectra of the DNA duplex exhibited a band with negative and positive peaks at approximately 245 and 275 nm, typical of B-DNA (**Figure S2A**). The CD spectra of the ACTD-DNA complexes showed a red shift from 275 to 287 nm, indicative of a conformational transition to an A-type structure. Moreover, no change was observed in the CD spectra of either DNA or the ACTD-DNA complex in the presence of spermine (**Figure S2B**). Thus, it is not possible that a spermine-induced conformational change of the DNA duplex affects the binding of ACTD to DNA.

### Spermine attenuates the inhibition of transcription and DNA replication by actinomycin D in vitro

**Figure 4:**
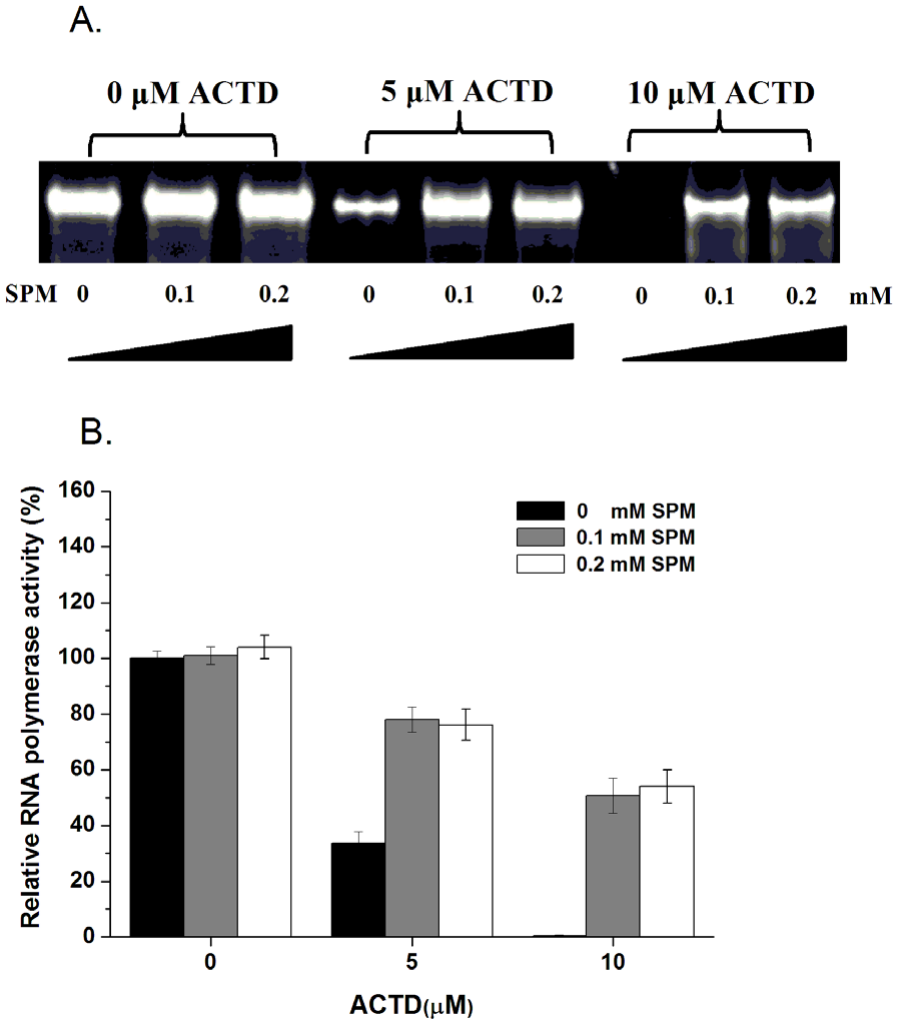
Spermine attenuates the inhibition of transcription by actinomycin D in vitro. (**A**) The effect of ACTD on T7 RNA polymerase activity in the presence of various concentrations of spermine (SPM). (**B**) Quantification of the percentage of RNA polymerase activity relative to the control treated with or without ACTD in the presence of various concentrations of spermine (SPM) (0, 0.1, and 0.2 mM). The data represent the mean values ±SDs from three separate experiments.

To explore whether spermine attenuates the inhibitory effects of ACTD on transcription *in vitro*, pTRI-β-actin-mouse cDNA was used as a template and treated with T7 RNA polymerase to monitor transcription in the presence of increasing concentrations of ACTD and spermine. T7 polymerase is a good model for *in vitro* transcription due to its extreme promoter specificity [40]. In the absence of ACTD, a 245-bp β-actin mRNA product was produced by T7 RNA polymerase at the same levels in the presence of increasing concentrations of spermine (Figure 4A). However, in the presence of increasing concentrations of ACTD (5 and 10 µM), the level of the RNA product was diminished (Figure 4A). Thus, the synthesis of RNA molecules by the T7 RNA polymerase was totally inhibited by ACTD at 10 µM (Figure 4A). Moreover, the presence of spermine at 0.1 and 0.2 mM rescued ∼60% of the transcriptional activity of RNA polymerase treated with 10 µM ACTD (Figure 4B), suggesting that spermine attenuates the inhibition of transcription by ACTD *in vitro*.

**Figure 5:**
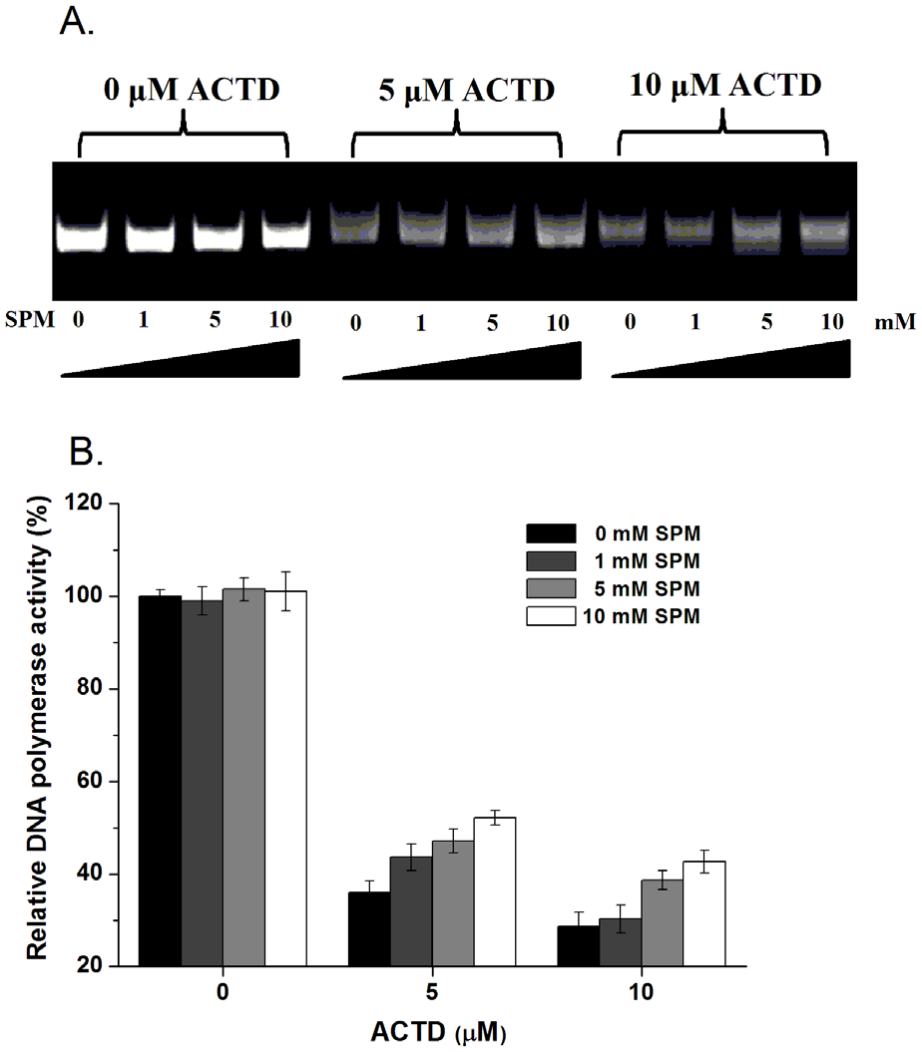
Spermine attenuates the inhibition of DNA replication by actinomycin D in vitro. (**A**) Effect of ACTD on *E. coli* DNA polymerase I activity in the presence of various concentrations of spermine (SPM). (**B**) Quantification of the percent RNA polymerase activity relative to the control treated with or without ACTD in the presence of various concentrations of spermine (SPM) (0, 1, 5, and 10 mM). The data represent the mean values ±SDs from three separate experiments.

To determine whether spermine attenuates the inhibitory effects of ACTD on DNA replication *in vitro*, a GC-rich fragment of the Cdc7 gene was used as a template and treated with *E. coli* DNA polymerase I in the presence of increasing concentrations of ACTD and spermine to monitor replication efficiency. In the absence of ACTD, DNA duplex molecules were synthesized by DNA polymerase I at the same level in the presence of increasing concentrations of spermine (Figure 5A). However, in the presence of increasing concentrations of ACTD (1, 5, and 10 µM), the level of DNA product was decreased, suggesting that ACTD inhibited DNA synthesis by the DNA polymerase (Figure 5B). Moreover, the presence of increasing spermine concentrations rescued the replication activity of DNA polymerase treated with ACTD, suggesting that spermine interferes with the inhibition of ACTD on DNA replication *in vitro*.

### Polyamine depletion enhances the inhibition of transcription and DNA replication by actinomycin D in cancer cell lines

**Figure 6:**
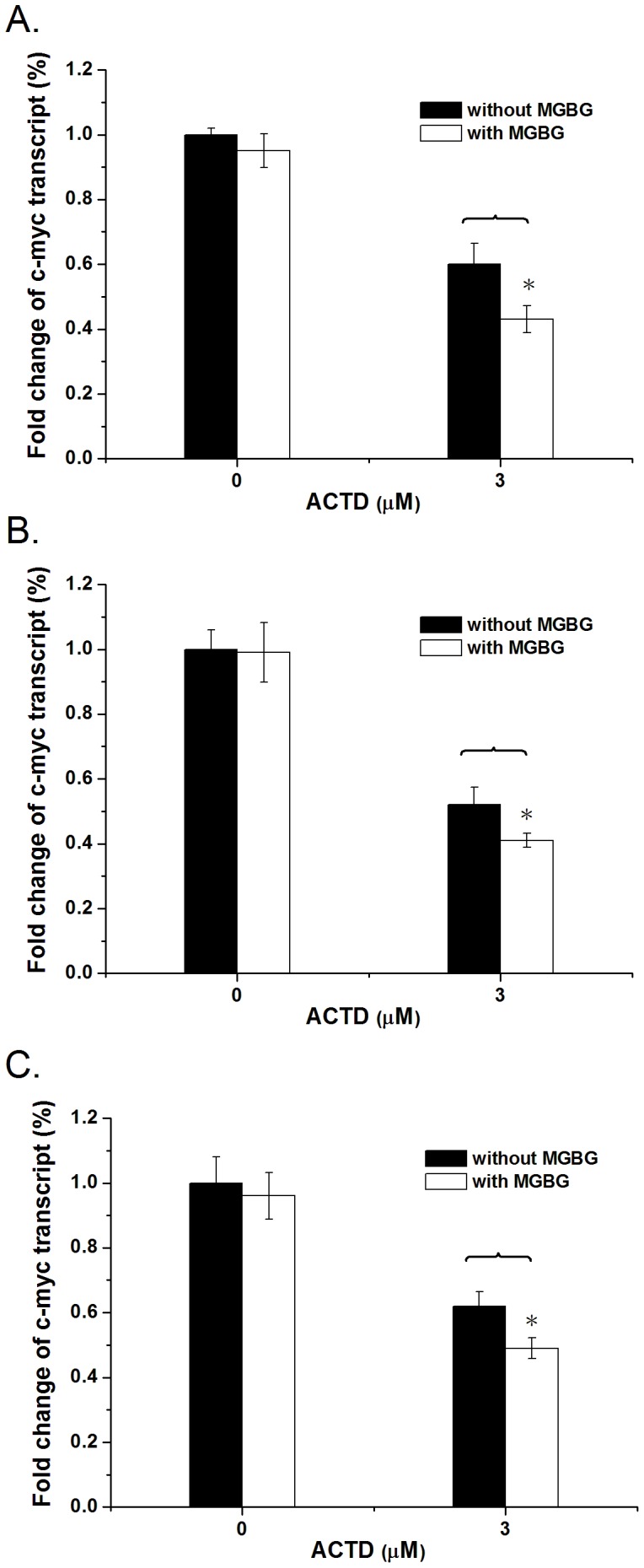
Polyamine depletion enhances the inhibition of transcription by actinomycin D within cells. The effects of ACTD at 3 µM on the fold change of *c-myc* gene expression in (**A**) HeLa, (**B**) A549, and (**C**) MCF7 cells by RT-PCR with and without a 24 h MGBG pretreatment versus control samples. β-actin was used as the internal control. The data represent the mean values ±SDs from three separate experiments (*p<0.05).

We showed that spermine attenuates DNA binding and the inhibition of transcription and DNA replication by ACTD *in vitro*. ACTD has been reported to inhibit the transcription of c-myc by binding to a GC sequence in the c-myc promoter [37]. To determine whether the inhibition of transcription by ACTD is affected by polyamines in cell models, HeLa, A549, and MCF7 cells were treated with ACTD after pretreatment with 2 µM MGBG, an inhibitor of polyamine synthesis [41]. We then monitored c-myc gene expression in cells using real time-PCR. The intracellular polyamine concentration was determined by dansylation method using TLC, and the intensity of polyamine dansylation was estimated to be reduced by 28, 23, and 25% upon treatment with 2 µM MGBG in HeLa, A549, and MCF 7 cells for 24 h, respectively, reflecting polyamine synthesis was inhibited by MGBG treatment for 24 hours in these cells (Figure S3). In addition, to determine whether the cancer cells can produce functionally polyamine in 6 h following ACTD treatment after polyamine depletion, intracellular polyamine concentration of MGBG-pretreated cancer cells without and with ACTD treatment for 6 h were determined and compared by dansylation method. The intensity of polyamine dansylation maintained at the same level in the presence or absence of ACTD, suggesting the cells may not produce functionally polyamine (including spermine) with ACTD treatment for 6 h after polyamine depletion (Figure S4). The expression of the c-myc gene was not affected by treatment with MGBG alone (2 µM) for 24 h in cancer cells (Figure 6). Cells cultured in the presence of 3 µM ACTD for 6 h displayed 0.6, 0.52 and 0.62 fold in c-myc expression of HeLa, A549, and MCF7 cells, respectively, indicating that ACTD inhibits c-myc expression in cancer cells (Figure 6). Moreover, MGBG pretreatment in the ACTD-treated HeLa, A549, and MCF7 cells markedly diplayed c-myc gene expression by 0.43, 0.41 and 0.49 fold in the presence of 3 µM ACTD, respectively, suggesting that depletion of the intracellular polyamine content increased the inhibitory activity of ACTD on the c-myc gene expression in cancer cells.

**Figure 7:**
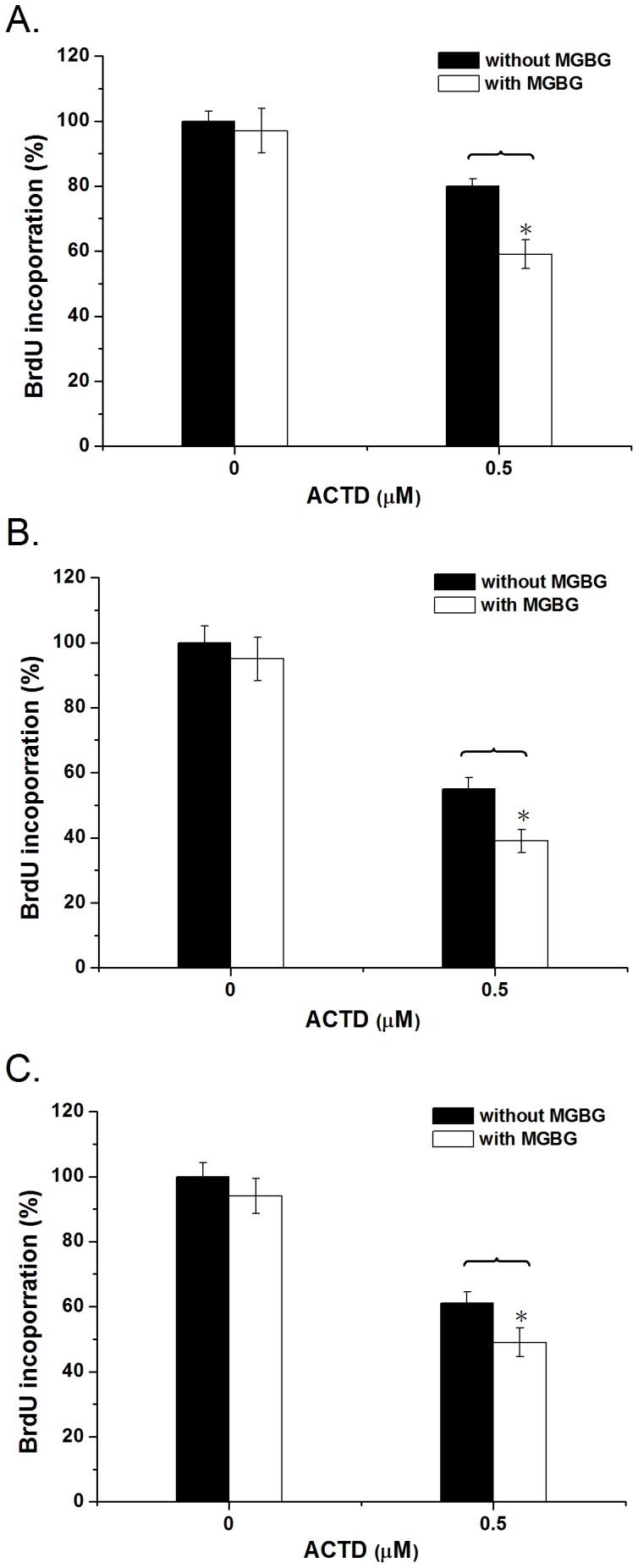
Polyamine depletion enhances the inhibition of DNA replication by actinomycin D within cells. The effects of ACTD at 0.5 µM on the relative BrdU incorporation in in (**A**) HeLa, (**B**) A549, and (**C**) MCF7 cells after a 24 h MGBG pretreatment versus control samples. The data represent the mean values ±SDs from three separate experiments (*p<0.05).

To determine whether the inhibition of DNA replication by ACTD is affected by polyamines in cancer cells, HeLa, A549, and MCF7 cells were treated with ACTD after pretreatment with 2 µM MGBG, and DNA synthesis was monitored using the BrdU incorporation assay. DNA synthesis was not affected by treatment with MGBG alone (2 µM) for 24 h (Figure 7). When ACTD was added to HeLa, A549, and MCF7 cells, BrdU incorporation appeared to be significantly decreased, indicating that ACTD inhibited DNA synthesis in the cells. Our results also showed that BrdU incorporation in HeLa, A549, and MCF7 cells treated with ACTD plus MGBG pretreatment was significantly lower than in those cells without MGBG pretreatment (Figure 7), implying that depletion of the intracellular polyamine content increases the inhibitory activity of ACTD on the DNA replication of the cancer cells.

To determine whether MGBG affect the transcription and DNA replication in normal cells, we used BEAS-2B cells (normal human lung cells) as a cell model to determine the c-myc transcription and DNA synthesis by treatment with MGBG for 24 h. The expression of the c-myc gene and DNA synthesis were not affected by treatment with MGBG alone (2 µM) for 24 h (Figure S5), showing MGBG does not affect the transcription and DNA replication in normal cells.

### Synergistic antiproliferative effect of polyamine inhibitor and actinomycin D

**Figure 8:**
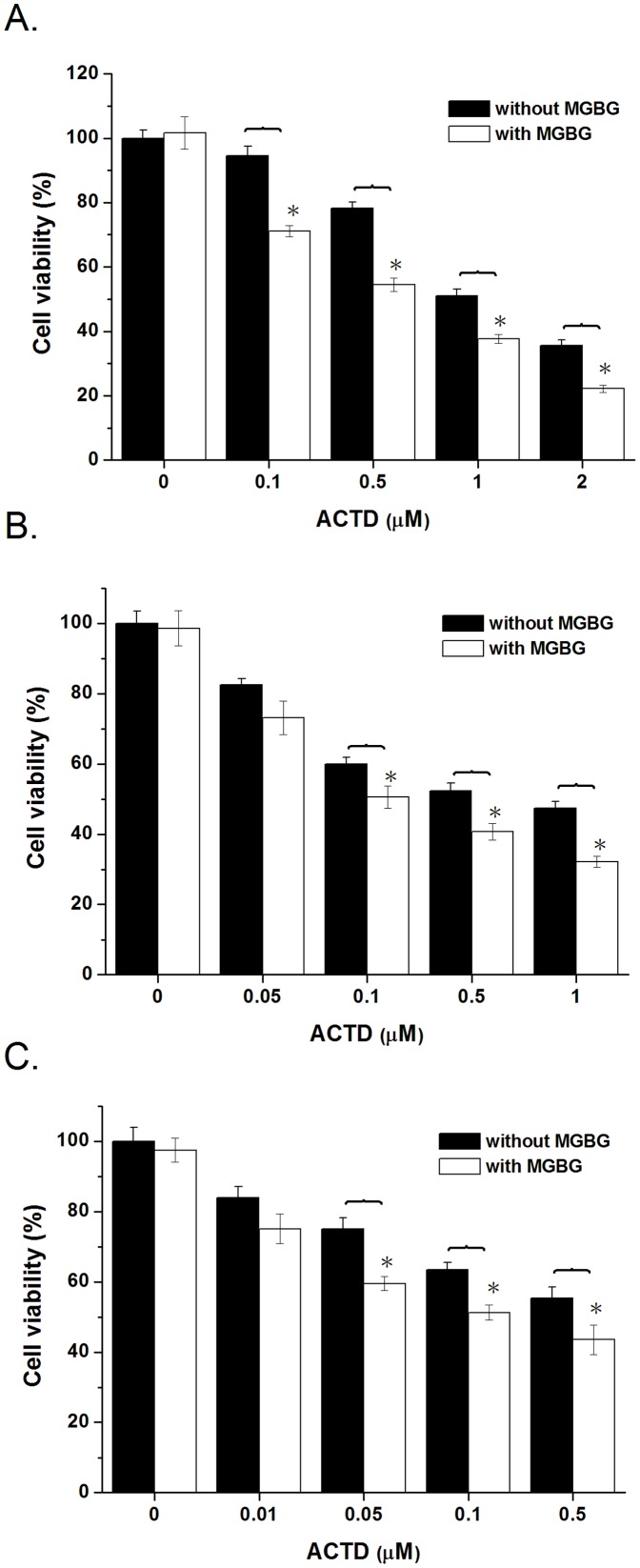
Polyamine depletion enhances the inhibition of cell viability by actinomycin D. The effects of ACTD at various concentrations on the viability of in (**A**) HeLa, (**B**) A549, and (**C**) MCF7 cells with or without a 24 h pretreatment with 2 µM MGBG. The data represent the mean values ±SDs from three separate experiments (*p<0.05).

The depletion of intracellular polyamine enhanced the inhibitory activities of ACTD on DNA replication and transcription, suggesting that a synergistic effect may be observed in the antiproliferative effects on cancer cells after treatment with a combination of polyamine inhibitor and ACTD. Here, we evaluated the effects of polyamine depletion on the application of ACTD to cancer therapy in HeLa, A549, and MCF7 cells. Cell viability was examined in the presence and absence of MGBG pretreatment (2 µM) and increasing concentrations of ACTD. Cell viability was not affected by pretreatment with MGBG alone in HeLa, A549, and MCF7 cells. Statistical analysis showed that cell viability was significantly decreased with increasing concentrations of ACTD in HeLa, A549, and MCF7 cells (Figure 8). Moreover, the addition of MGBG enhanced the cytotoxicity of ACTD at various concentrations in HeLa, A549, and MCF7 cells, suggesting a synergistic antiproliferative effect of the polyamine inhibitor and ACTD. This result implies that depleting the intracellular polyamine content increases the sensitivity of cancer cells to ACTD.

To determine the cytoxicity of MGBG toward normal cells, cell viability was assessed in BEAS-2B cells by treatment with MGBG for 24 h using an MTT assay, showing the IC_50_ value of BEAS-2B cells incubated with MGBG for 24 h was greater than 100 µM (Figure S6). These results suggest MGBG does not exert cytotoxicity in normal cells, but exhibits its potential for cancer therapy.

## Discussion

The interactions of cationic polyamines with the negatively charged phosphate groups of nucleic acids promote conformational changes in DNA structure, the condensation of DNA and chromatin, and the modulation of various aspects of gene replication, transcription, and translation [42]. In addition, spermine can protect DNA against damage caused by various mutagenic agents, including radiation and ROS [26]. Spermine may act by mechanisms that include the direct scavenging of ROS, the induction of conformational changes, and blocking DNA from interactions with mutagenic reagents [43], [44]. Currently, we are interested in the modulatory activity of polyamines on the activities of DNA intercalators. Using ACTD as a model, we have attempted to explore the effect of spermine on the DNA-associated properties of ACTD, including its DNA-binding activity and the inhibition of transcription and DNA replication. Our SPR results showed that the binding capacity of ACTD to hairpin DNA duplexes was attenuated by spermine. The binding of spermine with DNA results in the formation of a steric barrier that blocks the access of ACTD to the minor grooves of DNA [22], because spermine spans both the major and minor grooves of DNA duplexes with interstrand attachments [45]. We further investigated the kinetic behavior of ACTD bound to DNA by evaluating the SPR association and dissociation phases between the drug and DNA hairpins. The association phase mainly reflects the entry of drugs into the DNA grooves, while the dissociation phase measures the hydrogen bonding environment within the groove that accommodates the drugs [46]. We showed that the presence of spermine significantly decreases the rates of association between ACTD and DNA duplexes, while the dissociation of ACTD from the DNA duplexes is increased by spermine at various concentrations. These results suggested that spermine not only interferes with the access of ACTD to the minor groove but also affects the hydrogen bonding between ACTD and DNA, reflecting the critical role of polyamines in modulating the molecular recognition and interaction between the drug and its DNA partner. These results are further supported by the dramatic increases in the *T_m_* and Δ*G* of the DNA duplex observed upon ACTD binding; these increases in *T_m_* and Δ*G* are reduced in the presence of spermine.

T7 RNA polymerase and *E. coli* DNA polymerase I possess all of the fundamental features of eukaryotic RNA polymerases and DNA polymerases and serve as ideal model systems in which to study the functional mechanisms of transcription and replication *in vitro* [47], [48]. The present study provides evidence that ACTD is able to inhibit the transcriptional and replication activities of T7 RNA polymerase and DNA polymerase I, respectively, and that the inhibition is attenuated by the addition of spermine in a dose-dependent manner. Several mechanisms of the inhibition of transcription and replication *in vitro* by DNA-binding drugs have been proposed [49], [50]. The binding of ACTD with DNA could prevent RNA and DNA polymerase from binding to the DNA and affect the initiation of transcription and replication. In addition, the drug can block the progression of RNA polymerase and DNA polymerase along the template DNA to prematurely terminate transcription and replication. Our results suggest that spermine interferes with ACTD binding to the DNA template and reduces the inhibition of ACTD on replication and transcription. Moreover, spermine appears to increase the efficiency of RNA and DNA polymerases primarily through the stabilization of the enzyme-template initiation complex against the inhibitory effect of ACTD. Thus, polyamines appear to be potential candidates that could modulate the efficiency of this transcription and replication system. This finding is consistent with previous studies that showed that the concentration of natural polyamines increases when RNA transcription and replication proceed *in vivo* [51], [52]. In addition, polyamines were able to promote RNA transcription via the T7 RNA polymerase and DNA synthesis by DNA-dependent DNA polymerase *in vitro* [53], [54].

Because the regulation of the expression of the oncogene c-myc plays an important role in tumorigenesis, c-myc is a potential target for chemotherapy by DNA-binding drugs [55]. ACTD has been found to inhibit c-myc gene transcription by interacting with the c-myc promoter to block the access of its transcription factor and RNA polymerase [37]. MGBG has been applied as a potent competitive inhibitor of S-adenosylmethionine decarboxylase to decrease the spermine content of cancer cells [56]. We observed that decreasing intracellular polyamines by MGBG enhances the inhibition of ACTD on c-myc gene expression in cancer cells. In addition, the decrease in DNA synthesis caused by ACTD in polyamine-depleted cells was greater than that observed in cells without polyamine depletion. Consistent with the above *in vitro* results, polyamine depletion enhances the inhibition of DNA replication and transcription activity by ACTD in cancer cells, suggesting that polyamine attenuates the ACTD-dependent inhibition of DNA replication and transcription within cells.

We previously determined that polyamine depletion enhances the cytotoxicity of the dimeric mithramycin-Co(II) complex, a minor groove-binding drug [30]. In the present work, we observed that the combination of ACTD and MGBG also has a synergistic effect on the cytotoxicity of HeLa, A549, and MCF7 cells. The high concentration of the polyamine and its subsequent DNA-protecting activity in cancer cells decrease the anti-tumor activity of DNA-binding anticancer drugs [57]. In addition, the depletion of intracellular polyamines enhances either the efficacy of radiation therapy or the susceptibility of normal cells to oxidative stress [26]. Taken together, our biochemical and cell-based results suggest that polyamine plays a novel role in protecting cellular DNA from the DNA intercalator ACTD. This study should be of relevance to future studies aimed at developments in cancer therapy by enhancing the anticancer activity of this DNA intercalator though polyamine depletion.

## Supporting Information

Figure S1 **The melting curves of the DNA duplexes in the presence of increasing spermine with and without ACTD binding.**
(TIF)

Figure S2 **The CD spectra of the DNA duplex and ACTD-DNA complexes in the presence of various concentrations of spermine.**
(TIF)

Figure S3 **The polyamine contents of MGBG-pretreated HeLa, A549, and MCF7 cells.**
(TIF)

Figure S4 **The polyamine contents of MGBG-pretreated HeLa, A549, and MCF7 cells following ACTD treatment for 6 h.**
(TIF)

Figure S5 **The effects of polyamine depletion on the transcription and DNA replication in normal cells.** (**A**) The effects of MGBG at 2 µM on the fold change of *c-myc* gene expression. (**B**) The relative BrdU incorporation in BEAS-2B cells.
(TIF)

Figure S6 **The effects of polyamine depletion on the cell viability in normal cells.** The effects of MGBG at various concentrations on the viability of in BEAS-2B cells.
(TIF)
