# Correction: Spermine Attenuates the Action of the DNA Intercalator, Actinomycin D, on DNA Binding and the Inhibition of Transcription and DNA Replication

**DOI:** 10.1371/annotation/254cef67-ab0e-43f6-af62-8f7df7c3677c

**Published:** 2013-05-15

**Authors:** Sheng-Yu Wang, Alan Yueh-Luen Lee, Yi-Hua Lai, Jeremy J. W. Chen, Wen-Lin Wu, Jeu-Ming P. Yuann, Wang-Lin Su, Show-Mei Chuang, Ming-Hon Hou

The name of the second author should be: Alan Yueh-Luen Lee

The correct citation is: Wang S-Y, Lee AY-L, Lai Y-H, Chen JJW, Wu W-L, et al. (2012) Spermine Attenuates the Action of the DNA Intercalator, Actinomycin D, on DNA Binding and the Inhibition of Transcription and DNA Replication. PLoS ONE 7(11): e47101. doi:10.1371/journal.pone.0047101 

