# Supplementary figures and images for: Spermine Attenuates the Action of the DNA Intercalator, Actinomycin D, on DNA Binding and the Inhibition of Transcription and DNA Replication
Source: PLoS One. 2012 Nov 8;7(11):e47101. doi: 10.1371/journal.pone.0047101 (PMC3493566; doi:10.1371/journal.pone.0047101)

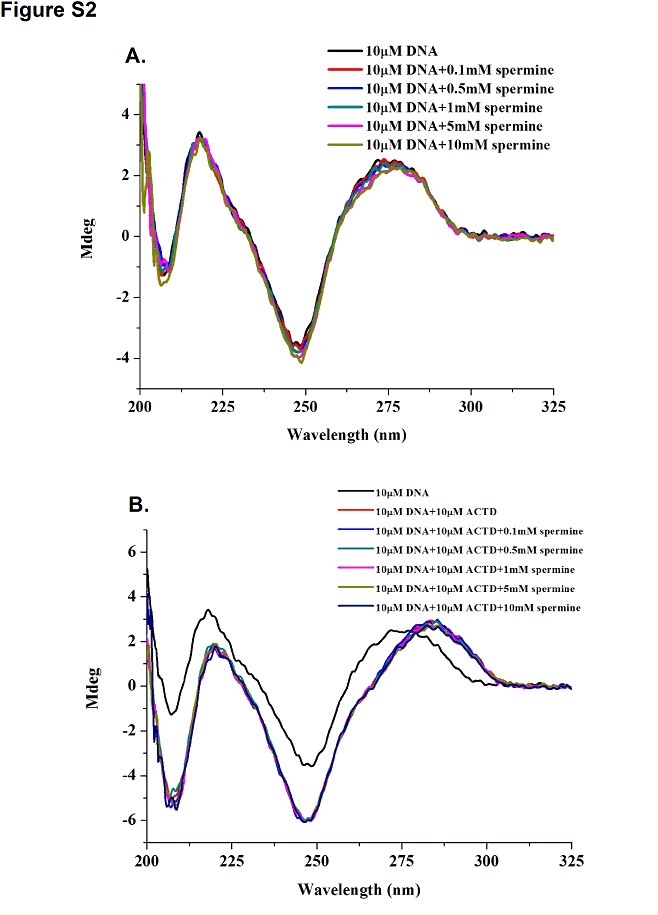

Supplement: Figure S2 — The CD spectra of the DNA duplex and ACTD-DNA complexes in the presence of various concentrations of spermine. (TIF) [file pone.0047101.s002.tif]
